# Behavioral and Biochemical Interaction Between Nicotine and Chronic Unpredictable Mild Stress in Mice

**DOI:** 10.1007/s12035-016-9701-0

**Published:** 2016-01-18

**Authors:** G. Biala, K. Pekala, A. Boguszewska-Czubara, A. Michalak, M. Kruk-Slomka, B. Budzynska

**Affiliations:** 1 Department of Pharmacology and Pharmacodynamics, Medical University of Lublin, Chodzki 4A Street, 20-093 Lublin, Poland; 2 Department of Medical Chemistry, Medical University of Lublin, Chodzki 4A Street, 20-093 Lublin, Poland

**Keywords:** Chronic mild stress, Nicotine, Oxidative stress, Anhedonia, Memory, Mice

## Abstract

Nicotine, the main component of tobacco smoke, exerts influence on mood, and contributes to physical and psychological dependence. Taking into account frequent concomitance of nicotine abuse and stress, we aimed to research behavioral and biochemical effects associated with nicotine administration in combination with chronic unpredictable mild stress (CUMS). Mice were submitted to the procedure of CUMS for 4 weeks, 2 h per day. Our results revealed that CUMS-exposed animals exhibited behavioral alteration like anxiety disorders in the elevated plus maze (EPM) test, the disturbances in memory in the passive avoidance (PA) test and depressive effects in the forced swim test (FST). Moreover, nicotine (0.05–0.5 mg/kg), after an acute or subchronic administration decreased stress-induced depression- and anxiety-like effect as well as memory deficit. Administration of metyrapone (50 mg/kg), a glucocorticosteroid antagonist, alleviated the depressive effect induced by the CUMS. The biochemical experiments showed decreased values of the total antioxidant status (TAS), activities of superoxide dismutase (SOD) and glutathione peroxidase (GPx) with simultaneously increased in malondialdehyde (MDA) concentration in mice submitted to the CUMS. The same effects were observed after an acute and subchronic nicotine administration within all examined brain structures (i.e., hippocampus, cortex, and cerebellum) and in the whole brain in non-stressed and stressed mice confirming pro-oxidative effect of nicotine. Our study contributes to the understanding of behavioral and biochemical mechanisms involved in stress-induced disorders such as depression, anxiety and memory disturbances as well as dual nicotine-stress interactions on the basis of the development of nicotine dependence.

## Introduction

Stress is related to mental and neurobiological disturbances [1] and can be described as a state of an organism characterized by an increase in emotional tension caused by threatening factors. Stress can be also defined as a process by which environmental factors (i.e., stressors) disturb the balance whereby the organism reacts to the threat. Internal stressors mostly result from disturbances of body homeostasis, external stressors most frequently result from functioning in particular social conditions. Stressors can activate complex mechanisms of mental and physiological reactions and to a large extent affect the condition of health of an individual.

Concerning the possible mechanisms involved, the sympathetic-adrenal system and the hypothalamus-pituitary-adrenals axis (HPA) are mostly responsible for the response of an organism to stress reactions [2]. The sympathetic system is activated immediately after the occurrence of a stressor causing an activation of the adrenal medulla and secretion of catecholamines, adrenaline and noradrenaline. These hormones are responsible for stimulation of the organism (increased blood flow to active muscles, intensified mental and physical activity, increased reaction of the circulatory system, intense breathing) resulting in a faster reaction to a danger. However, secreted in great amounts for a long time, they may disturb normal functioning of the organism and lead to its exhaustion [3].

The HPA plays a significant role in adaptive processes to stressful conditions [2, 3]. The effects of the HPA axis stimulation appear after several hours and may last even a few days. Activity of the HPA system is regulated by numerous neurotransmitter pathways, among others glutamatergic, ɣ-amino butyric (GABA)ergic, serotoninergic, noradrenergic and cholinergic. The HPA axis coordinates and controls secretion of glucocorticosteroids from the adrenal cortex to the blood [2]. Disturbances in the HPA axis functioning occur in many mental diseases, such as, among others, depression and chronic fatigue syndrome. As a result of prolonged activation of the HPA axis, glucocorticosteroids lead to neuronal damage of the hippocampus and frontal cortex, the structures responsible for emotional reactions. It has been shown that a high level of glucocorticosteroids in the blood can damage numerous dopaminergic, glutamatergic and serotoninergic neurons and also can lead to inhibition of the process of neurogenesis. All these changes subsequently result in a reduction of the hippocampus and frontal cortex volumes, which is characteristic of patients with severe depression [2, 4].

Research on pathogenesis of depression, its frequency and serious consequences is still continued. The use of animal models facilitates understanding of pathogenesis, etiology, and symptomatology of depression, examination of many genetic and epigenetic factors leading to this disease and searching for new and effective strategies of its treatment [5]. So far, numerous attempts to create animal models of depression have been made, which enable us to focus on at least certain aspects of the disease [6]. Models of depression are conducted in order to put animals without depression as closely as possible to the clinical situation.

Many different models of depression are used in the research conducted at present, including learned helplessness, forced swim test, or social defeat stress [4, 7]. However, the chronic unpredictable mild stress (CUMS) model is the most frequently used and considered one of the most perfect models of depression [4, 6]. Its aim is the induction of the state of anhedonia, which is the main symptom of depression in humans, by subjecting animals to the action of mild stress stimuli [8, 9]. In this model, long-term exposure of experimental animals to various mild unpredictable stressors (i.e., restriction, inversion of the light-darkness cycle, deprivation of water or food, wet litter) is related to significant changes in their behavior. The stimuli which initiate a response to stress, the so-called stressors, usually acting for 2 to 3 weeks, are potentially harmful to the organism and cause acute or chronic physiological reaction to stress [10]. All the behavioral changes induced in animals in this model can be reversed by administration of antidepressants [11].

In the first series of experiments, we aimed to evaluate the relationship between effects of an acute or subchronic nicotine administration and the CUMS in mice using different animal paradigms. In the context of the present study it should be added that, in the search for psychological sources of addiction, particular attention is paid to the role of stress and some common mechanisms of both phenomena. It has been suggested that stress plays a significant role not only in the genesis of addiction but also in maintenance of abstinence [12]. As such, stress is one of the main risk factors in the development of addictions and their recurrence. For instance, it has been found that people’s exposure to stressors increases the number of cigarettes smoked, the urge to smoke and the volume of inhaled tobacco smoke [13, 14]. Thus, the relationship between stress and effects of nicotine is not fully coherent and understood. Nicotine, as the main component of tobacco smoke, influences, through central mechanisms, the mood and emotional tension, and also contributes to development of physical and mental dependence. These effects, like in the case of other addictive agents, mostly involve dopaminergic neurons in the mesolimbic system which is a part of the reward system. Cigarette smoking, involving delivery of subsequent amounts of nicotine, causes a subjective feeling of pleasure and becomes a way of dealing with stress in smokers [15]. In certain experimental animal models, it has been demonstrated that chronic and acute stress may aggravate both behavioral as well as neuronal effects caused by administration of nicotine. The data also report that nicotine modifies the influence of stress on anxiety behavior and cognitive processes [16]. Taking into consideration frequent concomitance of nicotine abuse and stress which accompanies daily life, finding actual effects of simultaneous exposure to these factors can have great clinical and toxicological significance.

In the second series of biochemical experiments, we aimed to describe alterations in antioxidant barrier of the brain and its selected regions (i.e., prefrontal cortex, cerebellum and hippocampus) in a mouse model of CUMS with and without acute or subchronic nicotine administration. The presence of oxidative stress was validated by the level of tissue total antioxidant status (TAS), activities of some key antioxidant enzymes, like superoxide dismutase (SOD) and glutathione peroxidase (GPx) as well as concentration of malondialdehyde (MDA), the main product of lipid peroxidation.

In this context, it has been suggested that up-regulation of the stress hormones, induced by hyperactivation of HPA axis, may induce oxidative stress with the overproduction of reactive oxygen species (ROS) [17]. First of all, cortisol increases oxygen supply to target tissues (including brain) by increasing blood pressure and heart rate. Therefore more oxygen, which is delivered to all tissues, exerts more deleterious effects within cells. Brain is particularly vulnerable to oxidative stress. Weighting 2 % of total body mass it consumes over 20 % of oxygen used by the whole organism. Moreover, it consists of abundant polyunsaturated fatty acids that are substrates for ROS [18], and compounds easily undergoing redox reactions like iron ions or ascorbic acid [19]. Secondly, cortisol disturbs electron transport chain by destroying enzymes constituents of certain mitochondrial enzyme complex activities (NADH dehydrogenase—complex I, succinate dehydrogenase—complex II, cytochrome c reductase—complex III, cytochrome c oxidase—complex IV) and causing electron leakage. These phenomena liberate free electrons that form reactive species with neutral molecules. Although free radicals at physiological concentrations exert important functions in proper cell functioning, their increased amount may disturb main cellular processes. All cells have special emergency system, which is activated in case of increased free radicals level. The so-called antioxidant barrier consists of two systems: enzymatic and non-enzymatic ones. Antioxidant enzymes cooperate with each other to inhibit excessive production of ROS and to protect against their harmful action. SOD, a key antioxidant enzyme, catalyzes the reaction of superoxide anion radical (O-2) dismutation into oxygen and hydrogen peroxide. Then reduction of hydrogen peroxide to oxygen and water can occur in two different ways with participation of two different enzymes: GPx and catalase (CAT). GPx catalyzes the reaction with expense of glutathione, which is used as electron donor to regenerate the enzyme [20]. Non-enzymatic antioxidant barrier constituents, e.g., ascorbate, coenzyme Q10, vitamin E, and glutathione, are radical scavengers. They directly react with free radicals and detoxify them by removing their radical character throughout electron donation [21]. As the organism developed numerous mechanisms to protect oxidative homeostasis, some internal or external conditions (in form of acute or chronic stimuli) may disturb the equilibrium, causing oxidative stress. In such a situation, ROS attack main cellular constituents, like DNA, proteins or lipids, to destroy them. Oxidative stress induces lipid peroxidation, which destabilizes membranes and may cause cell death in every brain region [17]. The level of lipids peroxidation can be monitored by the concentration of the end product of the process, i.e., MDA level.

In total, both series of our complementary experiments for the first time aimed to evaluate behavioral and biochemical impact and complex relationship between nicotine and the CUMS, critical for the development and maintenance of nicotine dependence.

## Materials and Methods

### Ethics Statement

All experiments were conducted according to the National Institute of Health Guidelines for the Care and Use of Laboratory Animals and to the European Community Council Directive for the Care and Use of Laboratory Animals of 24 November 1986 (86/609/EEC). The protocol was approved by the Committee on the Ethics of Animal Experiments of the Medical University of Lublin (Permit Number: 43/2013). All efforts were made to minimize animal suffering and to reduce the number of animals used.

### Animals

The experiments were carried out on naive male Swiss mice (Farm of Laboratory Animals, Warsaw, Poland) weighing 20–25 g at the beginning of the experiments. The animals were maintained under standard laboratory conditions (12 h light/dark cycle, room temperature 21 ± 1 °C) with free access to tap water and laboratory chow (Agropol, Pulawy, Poland) and were adapted to the laboratory conditions for at least 1 week. Each experimental group consisted of 8–12 animals. Different mice were used for each drug and time treatment.

### Drugs

The following compounds were tested: (−) nicotine hydrogen tartrate (0.05, 0.1, 0.2 and 0.5 mg/kg, Sigma-Aldrich, St. Louis, MO, USA), and metyrapone (50 mg/kg, Tocris Bioscience, UK). Drugs were dissolved in saline solution (0.9 % NaCl). Nicotine was administered subcutaneously (s.c.) whereas metyrapone was administered intraperitoneally (i.p.) at a volume of 10 ml/kg. Drug doses refer to the salt form. The pH of the nicotine solution was adjusted to 7.0. Fresh drug solutions were prepared on each day of experimentation. Control groups received saline injections of the same volume and via the same route of administration.

The range of doses of drugs was chosen based on literature data [22, 23], our recently published articles [21, 24] and preliminary studies.

### Experimental Protocols

Mice subjected to the CUMS procedure (further described in details) were called as stressed mice. Unstressed mice were exposed to behavioral tests, and not subjected to the CUMS. Nicotine was administered 30 min before behavioral test and/or chronically 30 min before CUMS procedure to stressed as well as to unstressed control mice. Metyrapone was administered 60 min before behavioral tests. Behavioral testing was done in independent groups of mice on the 28th day.

At the beginning of the experiments, mice were randomly divided into different groups (8–12 mice in each group). Group I consisted of unstressed control and saline/nicotine/metyrapone administered mice (acutely, on the 28th day); group II comprised unstressed control mice administered saline or nicotine subchronically (once a day, on the 15–27th day and on the test day); group III consisted of stressed control and saline/nicotine/metyrapone administered mice (acutely, on the 28th day); group IV consisted of stressed control mice administered saline or nicotine subchronically (once a day, on the 15–27th day and on the test day).

#### CUMS Procedure

The CUMS protocol was performed as described previously [8, 25, 26] with minor modifications. In brief, mice were subjected to different kinds of mild stressors, which varied from day to day to make the stress procedure unpredictable. These stressors were randomly scheduled over a 1-week period and repeated throughout the 4 weeks experiment for 2 hs daily. There were a total of seven stressors: (1) lack of litter; (2) cage shaking; (3) lights on overnight; (4) damp sawdust overnight; (5) food deprivation overnight; (6) an electric buzzer, 90 dB for 5 min; (7) tilted cage at 45°. Non-stressed mice were left undisturbed in their home cages. Twenty-four hours after the end of the CUMS protocol, all animals were exposed to one of the behavioral paradigms described below.

#### Forced Swim Test

The forced swim test (FST) was as described by Porsolt et al. [7]. In brief, each mouse was placed individually in a glass cylinder (height 25 cm, diameter 10 cm) containing 10 cm of water at 23–25 °C. Mice were placed in the water and forced to swim for 6 min. The duration of immobility was recorded during the last 4 min of the 6 min test. A mouse was considered to be immobile when it stopped struggling and passively moved to remain floating and keep its head above water. Water was changed between trials and temperature was maintained at 23–25 °C. Immediately after the test, mice were covered by a dry towel and then placed under a heating lamp until they were dry.

#### Elevated Plus Maze Test

The experimental apparatus was shaped like a *plus* sign and consisted of a central platform (5 × 5 cm), two open arms (30 × 5 cm) opposite to each other and two equal-sized enclosed (30 × 5 × 15 cm) arms opposite to each other. The maze was made of dark Plexiglas, elevated to a height of 50 cm above the floor and illuminated by a dim light.

The used procedure was chosen based on our recently published data [21, 24] and to the method of Lister [27]. Anxiolytic activity was indicated by an increase in time spent on the open arms or in number of entries to the open arms; anxiogenic effects were characterized by a decrease in those measures. The percentage of time spent on the open arms was calculated, just as was the percentage of entries into the open arm. Additionally, the number of enclosed arm entries was recorded as the indicator of motor activity of tested animals.

#### Passive Avoidance Test

The apparatus and used procedure was described in detail in our previous article [21]. The apparatus consisted of two-compartment acrylic box with a lighted and darkened one. The light chamber was illuminated by a fluorescent light (8 W) and connected to the dark chamber which was equipped with an electric grid floor. Entrance of animals to the dark box was punished by an electric foot-shock (0.2 mA for 2 s). On the 1st day of training (pre-test), mice were placed individually into the light compartment and allowed to explore the light box. After 30 s, the guillotine door was raised to allow the mice enter the dark compartment. When the mice entered this dark compartment, the guillotine door was closed and an electric foot-shock (0.2 mA) of 2 s duration was immediately delivered. The latency time for entering the dark compartment was recorded (TL1).

In the subsequent trial (retention) 24 h later, the same mouse was again placed individually in the light compartment of the apparatus and the time taken to re-enter the dark compartment was recorded (TL2). No foot-shock was applied in this trial. The experimental procedure involved examination of memory acquisition (the animals received injections of the substance before pre-test) [28, 29].

It should be noted that doses of nicotine, i.e., active dose causing an antidepressant effect in the FST, inactive dose in the elevated plus maze (EPM) and procognitive dose (after an acute administration) in the passive avoidance (PA) test have been chosen according to the literature data and our previous experiments [21, 24, 29–31]. For subchronic injections, doses of nicotine have been slightly lower as compared to those administered acutely.

### Collection of Tissues

Immediately after the behavioral tests, mice were sacrificed by decapitation and the whole brain was carefully taken out and rinsed in ice-cold saline to remove blood. The cerebellum, cerebral cortex, and hippocampus were rapidly dissected. The whole brain as well as isolated structures was used for the study.

#### Preparation of Brain Homogenates

The collected tissues were homogenized in 10:1 (vol:wt) chilled Tris buffer (pH 7.4) on ice for 20 s and centrifuged at 10000 g for 10 min at 4 °C to separate nuclear debris. The supernatant was collected and used for further study. TAS, activity of SOD and GPx as well as MDA level were determined from these supernatants spectrophotometrically with use of HITACHI 2800 apparatus and microplate reader EPOCH.

#### Determination of MDA Concentration

Lipid peroxidation was analyzed by determination of MDA concentration using thiobarbituric acid (TBA) reaction [32]. Briefly, 0.5 ml of tissue homogenate supernatant was mixed with 2.5 ml 1.22 M TCA in 0.6 M HCl and allowed to stand for 15 min. Then 1.5 ml of 0.9 % TBA was added and the mixture was incubated for 30 min in a boiling water bath. After cooling, 4 ml of *n*-butanol was added and the mixture was shaken variously. The samples were centrifuged at 1500×*g* for 10 min and then the absorbance of organic phase was measured at 532 nm with respect to blank (*n*-butanol alone). The concentration of MDA was read from the standard curve obtained by using malonaldehyde bis-dimethylacetal and expressed as μM of MDA/g of wet tissue.

#### Determination of TAS

TAS of brain homogenates was determined with ready-to-use diagnostic kit TAS by RANDOX (Randox Laboratories Ltd., UK). The method assumes that ABTS^®^ (2,2′-Azino-di-[3-ethylbenzthiazoline sulphonate]) produce a radical cation ABTS^®*+^ when incubated with a peroxidase (metmyoglobin) and H_2_O_2_. The radical cation has a relatively stable blue-green color, however its production can be suppressed by the addition of antioxidants present in the examined samples. Changes in absorption measured at 600 nm are proportional to the antioxidant concentration in the tissues homogenates. Results are expressed in mmol/l tissue.

#### Determination of SOD Activity

The activity of SOD was measured with the use of ready-to-use diagnostic kits RANSOD by Randox. The method employs xantine and xantine oxidase (XOD) to generate superoxide radicals, which react with iodonitrotetrazolium chloride to form red formazan dye. The superoxide dismutase activity is then measured by the degree of inhibition of the reaction. The increase in absorbance at 505 nm is read. Results are expressed in U/g protein.

#### Determination of GPx Activity

The activity of GPx was measured with the use of ready-to-use diagnostic kits RANSEL by Randox. This method is based on that of Paglia and Valentine [33]. GPx catalyzed the oxidation of glutathione (GSH) by cumene hydroperoxide. In the presence of glutathione reductase (GR) and NADPH, the oxidized glutathione (GSSG) is immediately converted to the reduced form with a concomitant oxidation of NADPH to NADP+. The decrease in absorbance at 340 nm is measured. Results are expressed in U/g protein.

#### Determination of Protein Content

The protein content was determined by the Bradford method [34] using BSA as the standard.

### Statistical Analysis

The data were expressed as the means ± standard error of the mean (SEM). The statistical analyses were performed by the one-way and two-way analysis of variance (ANOVA). Post hoc comparison of means was carried out with the Tukey’s test for multiple comparisons, when appropriate. The confidence limit of *P* < 0.05 was considered statistically significant.

For the memory-related responses, the changes in PA performance were expressed as the difference between retention and training latencies and were taken as a latency index (IL). IL was calculated for each animal and reports as the ratio:

$$ \mathrm{I}\mathrm{L}=\left(\mathrm{TL}2-\mathrm{TL}1\right)/\mathrm{TL}1 $$

TL1—the time taken to enter the dark compartment during the training

TL2—the time taken to re-enter the dark compartment during the retention test.

All statistical tests were performed using GraphPad Prism version 5.01 for Windows (GraphPad Software, USA).

## Results

### Depression-like Behavior in Unstressed and Stressed Mice in the FST; Effects of Nicotine and Metyrapone

**Fig. 1:**
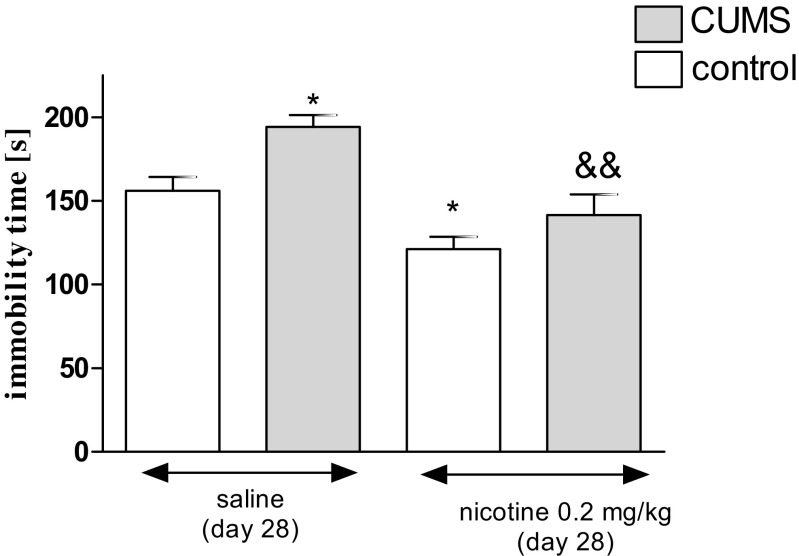
Effect of nicotine administered acutely in unstressed and stressed mice subjected to the CUMS in the FST. Nicotine (0.2 mg/kg) was administered s.c. 30 min before the test. The values represent the mean ± SEM (*n* = 8–12 mice per group). *P < 0.05 vs. unstressed, saline-treated group; &&*P* < 0.01 vs. stressed, saline-treated group (Tukey’s post hoc test)

Figure 1 describes the effect of an acute administration of nicotine in stressed (i.e., subjected to the CUMS protocol) and unstressed mice in the FST (two-way ANOVA: condition effect [*F*(1,41) = 11.05, *P* = 0.0019], treatment effect [*F*(1,41) = 25.01, *P* < 0.0001] without interaction effect [*F*(1,41) = 1.41, *P* = 0.3133]). A post hoc analysis showed that the exposition to the CUMS protocol increased immobility time in stressed mice as compared to unstressed control (*P* < 0.05). Furthermore, an acute treatment with nicotine (0.2 mg/kg) significantly decreased the immobility duration of stressed mice as compared with the stressed saline-treated group (*P* < 0.01). Nicotine also significantly reduced duration of swimming in unstressed mice as compared with the unstressed saline-treated group (*P* < 0.05).

**Fig. 2:**
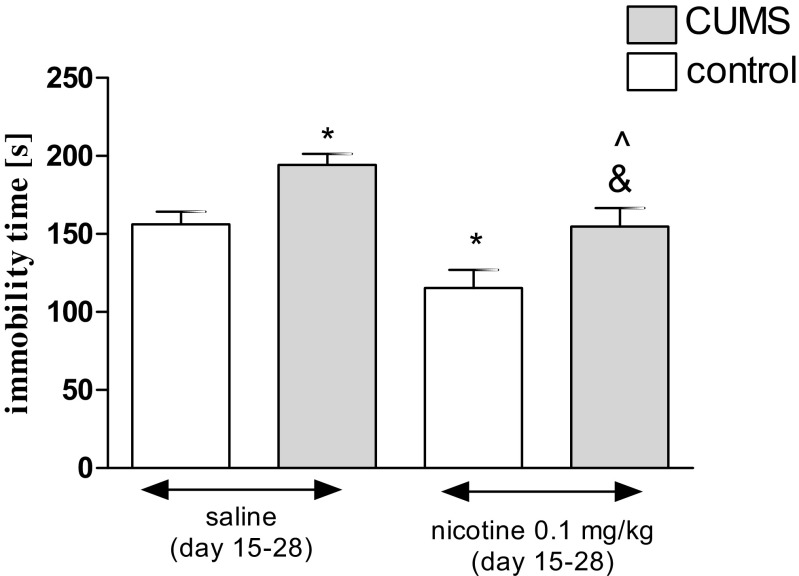
Effect of nicotine administered subchronically (day 15–27) in unstressed and stressed mice subjected to the CUMS in the FST. Nicotine (0.1 mg/kg) was administered s.c. 30 min before the CUMS procedure as well as to unstressed control mice and additionally 30 min before the FST (day 28). The values represent the mean ± SEM (*n* = 8–12 mice per group). *P < 0.05 vs. unstressed, saline-treated group, &*P* < 0.05 vs. stressed, saline-treated group; ^*P* < 0.05 vs. unstressed, nicotine-treated group (Tukey’s post hoc test)

Figure 2 shows the effect of a subchronic administration of nicotine in stressed and unstressed mice in the FST (two-way ANOVA: condition effect [*F*(1,37) = 16.46, *P* = 0.0002], treatment effect [*F*(1,37) = 17.58, *P* = 0.0002] without interaction effect [*F*(1,37) = 0.01, *P* = 0.9420]). A post hoc analysis further confirmed that the exposition to the CUMS protocol increased immobility time in stressed mice as compared with unstressed control (*P* < 0.05). Furthermore, repeated treatment with nicotine (0.1 mg/kg) significantly changed the immobility duration of stressed mice as compared to the stressed saline-treated group (*P* < 0.05) and with unstressed mice, after subchronic nicotine treatment (*P* < 0.05). Nicotine also significantly reduced duration of swimming in unstressed mice as compared with the unstressed saline-treated group (*P* < 0.05).

**Fig. 3:**
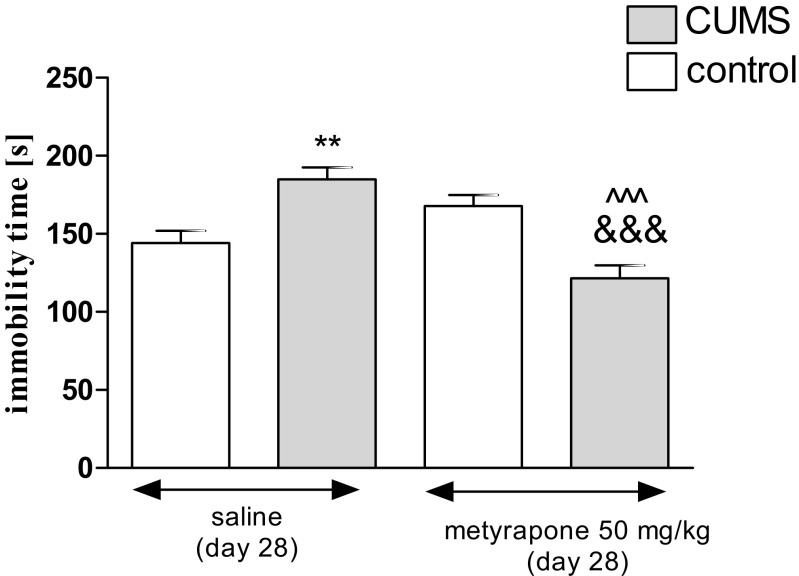
Effect of metyrapone in unstressed and stressed mice subjected to the CUMS in the FST. Metyrapone (50 mg/kg) was administered i.p. 60 min before the test. The values represent the mean ± SEM (*n* = 8–12 mice per group). **P < 0.01 vs. unstressed, saline-treated group; &&&*P* < 0.001 vs. stressed, saline-treated group; ^^^*P* < 0.001 vs. stressed, metyrapone-treated group (Tukey’s post hoc test)

Figure 3 presents the effect of an acute administration of metyrapone in stressed and unstressed mice in the FST (two-way ANOVA: treatment effect [*F*(1,41) = 73.40, *P* < 0.0001] and interaction effect [*F*(1,41) = 385.53, *P* < 0.0001] without condition effect [*F*(1,41) = 1.42, *P* = 0.2398]). A post hoc analysis further confirmed that the exposition to the CUMS protocol increased immobility time in stressed mice as compared with unstressed control (*P* < 0.01). Furthermore, an acute treatment with metyrapone (50 mg/kg) significantly decreased the immobility duration of stressed mice as compared with stressed, saline-treated group and unstressed, metyrapone-treated group (*P* < 0.001).

### Anxiety-like Behavior in Unstressed and Stressed Mice in the EMP; Effects of Nicotine

**Fig. 4:**
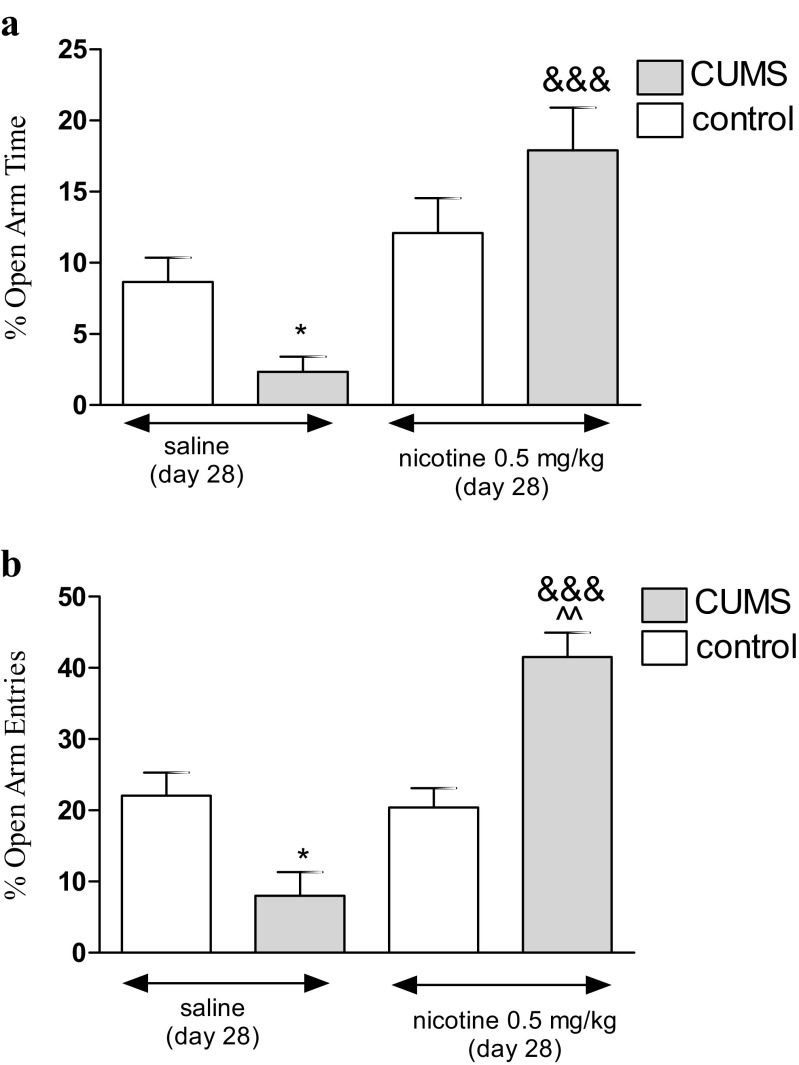
Effect of nicotine administered acutely in unstressed and stressed mice subjected to the CUMS on percentage of time spent in open arms (**a**) and percentage of open arm entries (**b**) in the EPM test. Nicotine (0.5 mg/kg) was administered s.c. 30 min before the test. The values represent the mean ± SEM (*n* = 8–12 mice per group). *P < 0.05 vs. unstressed, saline-treated group; &&&*P* < 0.001 vs. stressed, saline-treated group; ^^*P* < 0.01 vs. unstressed, nicotine-treated group (Tukey’s post hoc test)

**Fig. 5:**
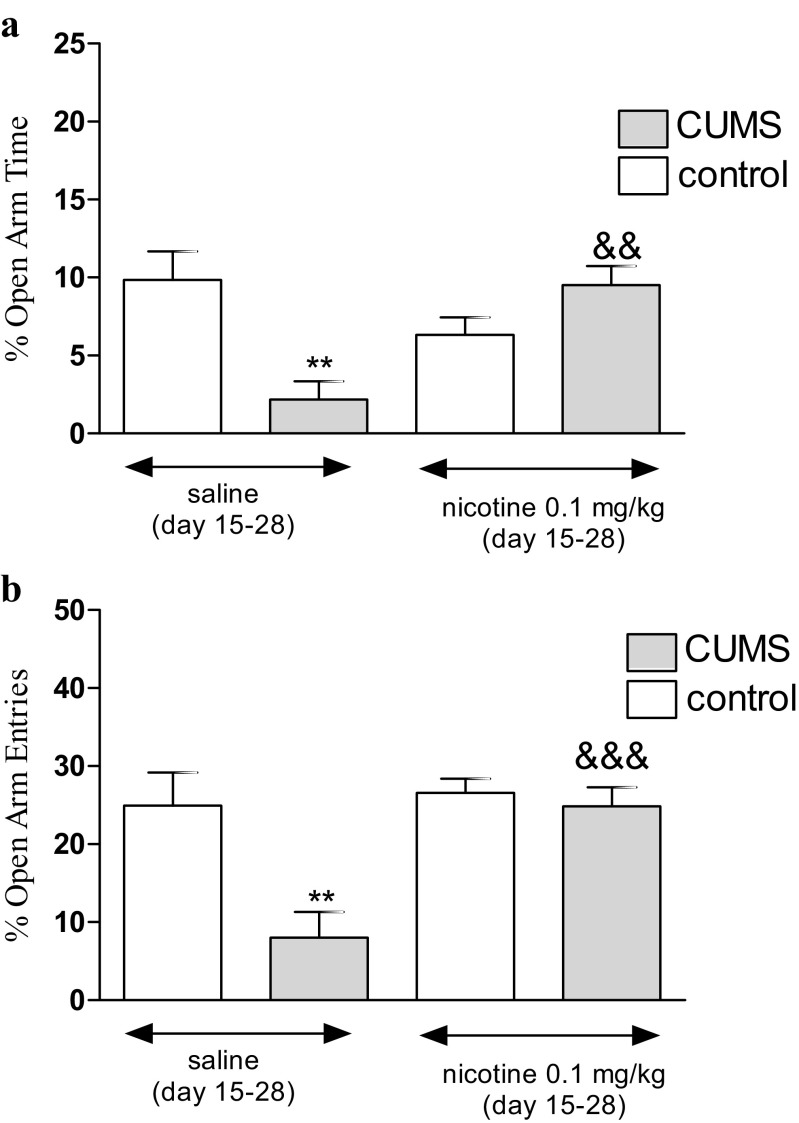
Effect of nicotine administered subchronically (day 15–27) in unstressed and stressed mice subjected to the CUMS on percentage of time spent in open arms (**a**) and percentage of open arm entries (**b**) in the EPM test. Nicotine (0.1 mg/kg) was administered s.c. 30 min before the CUMS procedure as well as to unstressed control mice and additionally 30 min before the EPM (day 28). The values represent the mean ± SEM (*n* = 8–12 mice per group). **P < 0.01 vs. unstressed, saline-treated group; &&*P* < 0.01 and &&&*P* < 0.001 vs. stressed, saline-treated group (Tukey’s post hoc test)

Figure 4 presents the effect of an acute administration of nicotine in stressed (i.e., subjected to the CUMS protocol) and unstressed mice on the percentage of time spent on the open arms (two-way ANOVA: treatment effect [*F*(1,26) = 81.47, *P* < 0.0001], interaction effect [*F*(1,26) = 33.23, *P* < 0.0001] without condition effect [*F*(1,26) = 0.06, *P* = 0.8017]) as well as the percentage of open arm entries (two-way ANOVA: condition effect [*F*(1,36) = 11.47, *P* = 0.0017], treatment effect [*F*(1,36) = 235.48, *P* < 0.0001] and interaction effects [*F*(1,36) = 278.49, *P* < 0.0001]). A post hoc analysis showed that the exposition to the CUMS protocol decreased the percentage of time spent on the open arms (Fig. 4a) and the percentage of open arm entries (Fig. 4b) in stressed mice as compared with unstressed control (*P* < 0.05). Furthermore, an acute treatment with nicotine (0.5 mg/kg) significantly increased both values in nicotine-treated stressed mice as compared with the control saline-treated group (*P* < 0.001) (Fig. 4a, b). The percentage of open arm entries was also increased in nicotine-treated stressed mice as compared with the nicotine-treated unstressed group (*P* < 0.01) (Fig. 5b). However, there was no influence on anxiety-like behaviors in nicotine-treated (0.5 mg/kg) unstressed mice.

Figure 5 shows the effect of a subchronic administration of nicotine in stressed and unstressed mice on the percentage of time spent on the open arms (two-way ANOVA: condition effect [*F*(1,40) = 27.72, *P* < 0.0001], treatment effect [*F*(1,40) = 20.16, *P* < 0.0001], with interaction effect [*F*(1,40) = 161.58, *P* < 0.0001]) as well as the percentage of open arm entries (two-way ANOVA: condition effect [*F*(1,41) = 103.72, *P* < 0.0001], treatment effect [*F*(1,41) = 102.39, *P* < 0.0001] with interaction effect [*F*(1,41) = 69.13, *P* < 0.0001]). A post hoc analysis showed that the exposition to the CUMS protocol decreased the percentage of time spent on the open arms (Fig. 5a) and the percentage of open arm entries (Fig. 5b) in stressed mice as compared with unstressed control (*P* < 0.01). Furthermore, subchronic treatment with nicotine (0.1 mg/kg) significantly increased the time spent on the open arms (*P* < 0.01) (Fig. 5a) and the percentage of open arm entries (*P* < 0.001) (Fig. 5b) in nicotine-treated stressed mice as compared with the control saline-treated group. However, there was no influence on anxiety-like behavior in nicotine-treated (0.1 mg/kg) unstressed mice.

Moreover, the CUMS protocol as well as an acute or subchronic nicotine injection to stressed and unstressed mice did not provoke any changes in number of enclosed arm entries in the EPM, thus causing no changes in the locomotor activity of animals (*P* > 0.05, post hoc Tukey’s test) (Table 1).

**Table 1:** Mean number (± SEM) of enclosed arms entries in the EPM test in stressed or unstressed mice treated acutely (A) or chronically (B) with nicotine; *n* = 8–12

A				
Treatment	CUMS	Unstressed control	CUMS+ nicotine 0.5 mg/kg (day 28)	Control + nicotine 0.5 mg/kg (day 28)
The number of enclosed arms entries	10.600 ± 1.087	8.500 ± 1.493	10.130 ± 0.581	8.286 ± 0.918
B				
Treatment	CUMS	Unstressed control	CUMS+ nicotine 0.1 mg/kg (day 14–27)	Control + nicotine 0.1 mg/kg (day 14–27)
The number of enclosed arms entries	11.020 ± 1.220	9.100 ± 1.236	11.230 ± 0.623	13.020 ± 0.7901

### Memory-Related Behavior in Unstressed and Stressed Mice in the PA Task; Effects of Nicotine

**Fig. 6:**
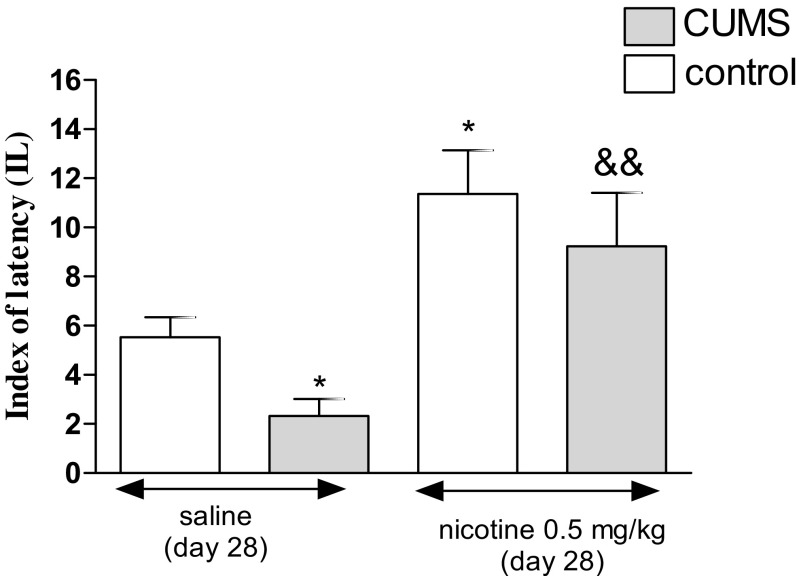
Effect of nicotine administered acutely in unstressed and stressed mice subjected to the CUMS in the PA task. Nicotine (0.5 mg/kg) was administered s.c. 30 min before the pre-test. The values represent the mean ± SEM (*n* = 8–12 mice per group). *P < 0.05 vs. unstressed, saline-treated group; &&*P* < 0.01 vs. stressed, saline-treated group (Tukey’s post hoc test)

Figure 6 presents the effect of an acute administration of nicotine in stressed (i.e., subjected to the CUMS protocol) and unstressed mice in the PA task (two-way ANOVA: condition effect [*F*(1,35) = 34.47, *P* < 0.0001], treatment effect [*F*(1,35) = 198.38, *P* < 0.0001] without interaction effect [*F*(1,35) = 1.41, *P* = 0.2423]). A post hoc analysis showed that the exposition to the CUMS protocol decreased IL value in stressed mice as compared with unstressed control (*P* < 0.05). Furthermore, an acute treatment with nicotine (0.5 mg/kg) significantly increased IL value in stressed mice as compared with the stressed saline-treated group (*P* < 0.01). Nicotine also significantly increased IL value in unstressed mice as compared with unstressed saline-treated group (*P* < 0.05).

**Fig. 7:**
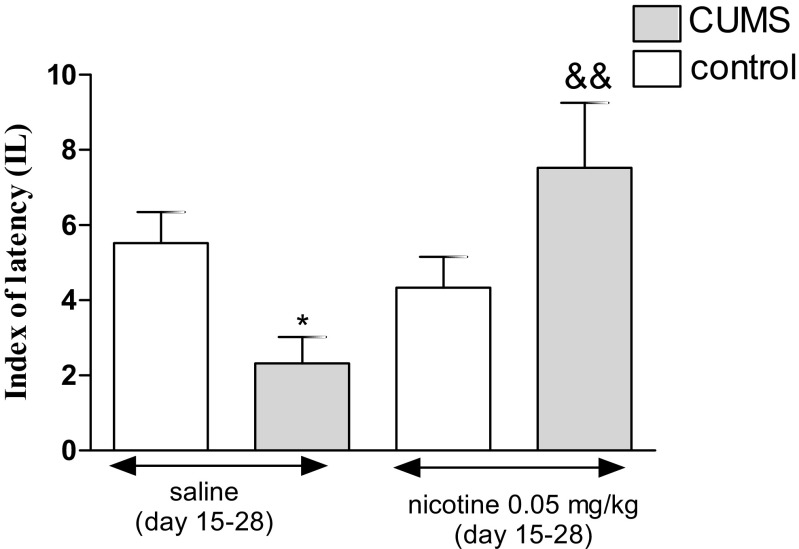
Effect of nicotine administered subchronically (day 15–27) in unstressed and stressed mice subjected to the CUMS in the PA task. Nicotine (0.05 mg/kg) was administered s.c. 30 min before the CUMS procedure as well as to unstressed control mice and 30 min before the PA test (day 28). The values represent the mean ± SEM (*n* = 8–12 mice per group). *P < 0.05 vs. unstressed, saline-treated group; &&*P* < 0.01 vs. stressed, saline-treated group (Tukey’s post hoc test)

Figure 7 shows the effect of a subchronic administration of nicotine in stressed and unstressed mice in the PA task (two-way ANOVA: treatment effect [*F*(1,38) = 39.23, *P* < 0.0001], interaction effect [*F*(1,38) = 99.22, *P* < 0.0001], without condition effect [*F*(1,38) = 0.00, *P* = 0.9889]). A post hoc analysis showed that the exposition to the CUMS protocol decreased IL value in stressed mice as compared with unstressed control (*P* < 0.05). Furthermore, subchronic treatment with nicotine (0.05 mg/kg) significantly increased IL value in stressed mice as compared with the stressed saline-treated group (*P* < 0.01). However, there was no influence of nicotine treatment on IL value in unstressed mice.

### Oxidative Stress Biomarkers in Brain and its Particular Structures in Unstressed and Stressed Mice; Effects of Nicotine

Table 2 shows effects of the CUMS and an acute administration of nicotine in stressed and unstressed mice on chosen markers of oxidative stress. Data are presented for TAS: in the whole brain (two-way ANOVA: condition effect [*F*(3,72) = 16.57, *P* < 0.0001], treatment effect [*F*(1,72) = 32.48, *P* < 0.0001] without interaction effect [*F*(3,72) = 0.41, *P* = 0.7494]) as well as in single structures as cerebellum (two-way ANOVA: condition effect [*F*(3,72) = 31.01, *P* < 0.0001], treatment effect [*F*(1,72) = 217.67, *P* < 0.0001] without interaction effect [*F*(3,72) = 0.51, *P* = 0.6800]), hippocampus (two-way ANOVA: condition effect [*F*(3,72) = 14.84, *P* < 0.0001], treatment effect [*F*(1,72) = 24.65, *P* < 0.0001] without interaction effect [*F*(3,72) = 1.70, *P* = 0.1754]), and cortex (two-way ANOVA: condition effect [*F*(3,72) = 17.67, *P* < 0.0001], treatment effect [*F*(1,72) = 20.62, *P* < 0.0001] without interaction effect [*F*(1,72) = 0.31, *P* = 0.8179]); SOD: in the whole brain (two-way ANOVA: condition effect [*F*(3,72) = 36.84, *P* < 0.0001], treatment effect [*F*(1,72) = 201.13, *P* < 0.0001] with interaction effect [*F*(3,72) = 4.08, *P* = 0.0098]) as well as in single structures as cerebellum (two-way ANOVA: condition effect [*F*(3,72) = 25.12, *P* < 0.0001], treatment effect [*F*(1,72) = 113.13, *P* < 0.0001] without interaction effect [*F*(3,72) = 1.47, *P* = 0.2298]), hippocampus (two-way ANOVA: condition effect [*F*(3,72) = 43.20, *P* < 0.0001], treatment effect [*F*(1,72) = 252.45, *P* < 0.0001] with interaction effect [*F*(3,72) = 6.19, *P* = 0.0008]), and cortex (two-way ANOVA: condition effect [*F*(3,72) = 30.23, *P* < 0.0001], treatment effect [*F*(1,72) = 316.74, *P* < 0.0001] with interaction effect [*F*(1,72) = 12.58, *P* < 0.0001]); GPx in the whole brain (two-way ANOVA: condition effect [*F*(3,72) = 37.59, *P* < 0.0001], treatment effect [*F*(1,72) = 321.08, *P* < 0.0001] with interaction effect [*F*(3,72) = 8.33, *P* < 0.0001]) as well as in single structures as cerebellum (two-way ANOVA: condition effect [*F*(3,72) = 43.29, *P* < 0.0001], treatment effect [*F*(1,72) = 927.89, *P* < 0.0001] with interaction effect [*F*(3,72) = 8.11, *P* < 0.0001]), hippocampus (two-way ANOVA: condition effect [*F*(3,72) = 7.49, *P* = 0.0002], treatment effect [*F*(1,72) = 198.87, *P* < 0.0001] with interaction effect [*F*(3,72) = 3.30, *P* = 0.0250]), and cortex (two-way ANOVA: condition effect [*F*(3,72) = 34.19, *P* < 0.0001], treatment effect [*F*(1,72) = 430.41, *P* < 0.0001] without interaction effect [*F*(1,72) = 1.61, *P* = 0.1946]); MDA in the whole brain (two-way ANOVA: condition effect [*F*(3,72) = 26.23, *P* < 0.0001], treatment effect [*F*(1,72) = 114.85, *P* < 0.0001] without interaction effect [*F*(3,72) = 0.50, *P* = 0.6839]) as well as in single structures as cerebellum (two-way ANOVA: condition effect [*F*(3,72) = 12.79, *P* = 0.0001], treatment effect [*F*(1,72) = 479.98, *P* < 0.0001] without interaction effect [*F*(3,72) = 0.75, *P* = 0.5268]), hippocampus (two-way ANOVA: condition effect [*F*(3,72) = 16.17, *P* < 0.0001], treatment effect [*F*(1,72) = 278.13, *P* < 0.0001] without interaction effect [*F*(3,72) = 3.12, *P* = 0.0311]), and cortex (two-way ANOVA: condition effect [*F*(3,72) = 17.05, *P* < 0.0001], treatment effect [*F*(1,72) = 17.03, *P* < 0.0001] without interaction effect [*F*(1,72) = 1.24, *P* = 0.3002]).

**Table 2:** Effect of the CUMS and nicotine administered acutely in unstressed and stressed mice subjected to the CUMS on chosen parameters of antioxidant barrier and process of lipids peroxidation. Nicotine (0.1, 0.2, and 0.5 mg/kg) was administered s.c. 30 min before the CUMS procedure as well as to unstressed control mice on 28th day of the experiment

	Tissue	Non-stressed				Stressed			
		Saline	Nic 0.1	Nic 0.2	Nic 0.5	Saline	Nic 0.1	Nic 0.2	Nic 0.5
TAS	Brain	1.843 ± 0.204	1.748 ± 0.206	1.616 ± 0.176	1.416 ± 0.160***	1.541 ± 0.161**	1.511 ± 0.190	1.431 ± 0.159	1.214 ± 0.189^##^
	Cerebellum	2.069 ± 0.211	1.967 ± 0.172	1.763 ± 0.154 ***	1.604 ± 0.115***	1.522 ± 0.172***	1.466 ± 0.128	1.282 ± 0.142^#^	1.171 ± 0.105^###^
	Hippocampus	1.565 ± 0.176	1.418 ± 0.155	1.333 ± 0.149*	1.206 ± 0.091***	1.296 ± 0.135**	1.280 ± 0.126	1.242 ± 0.076	1.094 ± 0.160^#^
	Cortex	1.630 ± 0.207	1.518 ± 0.186	1.420 ± 0.123	1.289 ± 0.117***	1.451 ± 0.143	1.413 ± 0.166	1.231 ± 0.190	1.100 ± 0.149^###^
SOD	Brain	2.939 ± 0.031	2.759 ± 0.397	2.562 ± 0.221	1.900 ± 0.236***	1.896 ± 0.334***	1.931 ± 0.289	1.709 ± 0.208	1.408 ± 0.113^##^
	Cerebellum	8.605 ± 0.987	8.178 ± 0.987	7.456 ± 0.870	6.191 ± 1.084***	6.875 ± 1.048**	5.213 ± 1.092^#^	5.140 ± 0.719^##^	4.098 ± 0.799^###^
	Hippocampus	14.71 ± 1.417	14.06 ± 1.460	11.24 ± 1.394***	8.853 ± 1.188***	9.091 ± 1.483***	7.696 ± 1.462	6.864 ± 1.498^#^	5.935 ± 0.803^###^
	Cortex	14.18 ± 1.443	13.44 ± 1.176	10.58 ± 1.632***	8.496 ± 1.067***	7.386 ± 1.515***	6.459 ± 1.426	6.149 ± 1.219	5.870 ± 0.787
GPx	Brain	17.14 ± 1.082	15.82 ± 2.246	13.50 ± 1.839***	10.18 ± 1.574***	9.524 ± 1.334***	8.296 ± 1.049	7.642 ± 1.183	6.765 ± 1.486^##^
	Cerebellum	34.86 ± 2.852	31.10 ± 2.799*	28.23 ± 2.620***	23.07 ± 2.176***	15.70 ± 2.564***	13.64 ± 2.193	12.64 ± 1.708	10.93 ± 1.661^###^
	Hippocampus	62.12 ± 4.902	59.75 ± 7.355	51.62 ± 5.470*	47.09 ± 6.954***	35.79 ± 3.795***	33.00 ± 5.299	31.63 ± 5.624	32.23 ± 12.58
	Cortex	66.54 ± 3.707	58.01 ± 5.553	51.90 ± 8.672***	44.88 ± 6.299***	35.81 ± 3.619***	30.44 ± 6.481	24.65 ± 5.607^##^	22.19 ± 5.119^###^
MDA	Brain	2.450 ± 0.654	3.150 ± 0.565	4.174 ± 1.415*	5.466 ± 1.502***	5.211 ± 0.857***	5.621 ± 0.901	7.377 ± 1.372	7.890 ± 1.354^###^
	Cerebellum	2.171 ± 0.456	2.329 ± 0.365	2.769 ± 0.497	3.177 ± 0.465*	5.633 ± 0.803***	5.788 ± 0.894	6.146 ± 0.957	6.551 ± 0.642
	Hippocampus	1.883 ± 0.269	1.946 ± 0.370	2.258 ± 0.341	2.830 ± 0.236***	3.317 ± 0.472***	3.359 ± 0.415	3.578 ± 0.257	3.695 ± 0.261
	Cortex	11.62 ± 0.778	15.75 ± 2.495*	18.12 ± 2.798***	19.38 ± 4.188***	16.38 ± 3.104**	18.53 ± 3.352	19.50 ± 2.490	21.36 ± 3.239^##^

The values represent the mean ± SEM (*n* = 8–12 mice per group). *P < 0.05, **P < 0.01, ***P < 0.001 vs. unstressed, saline-treated group, ^#^ *P* < 0.05, ^##^ *P* < 0.01, ^###^ *P* < 0.001 vs. stressed, saline-treated group (Tukey’s post hoc test)

A post hoc analysis showed that the exposition to the CUMS protocol decreased the values of TAS (whole brain *P* < 0.01, cerebellum *P* < 0.001, hippocampus *P* < 0.01), activities of SOD (whole brain *P* < 0.001, cerebellum *P* < 0.01, hippocampus *P* < 0.001, cortex *P* < 0.001) and GPx (whole brain *P* < 0.001, cerebellum *P* < 0.001, hippocampus *P* < 0.001, cortex *P* < 0.001) with simultaneous increased in MDA concentrations (whole brain *P* < 0.001, cerebellum *P* < 0.001, hippocampus *P* < 0.001, cortex *P* < 0.01) in stressed mice as compared with unstressed control.

Additionally, nicotine (0.5 mg/kg) decreased TAS value in stressed mice in the whole brain (*P* < 0.01), cerebellum (*P* < 0.001), hippocampus (*P* < 0.05), cortex (*P* < 0.001), whereas nicotine at the dose of 0.2 mg/kg decreased TAS value only in the hippocampus (*P* < 0.05) as compared with the stressed saline-treated group. Furthermore, in unstressed mice an acute treatment with 0.2 mg/kg of nicotine significantly increased TAS value in the cerebellum (*P* < 0.001) and hippocampus (*P* < 0.05), and after 0.5 mg/kg in the whole brain and all examined structures (*P* < 0.001) as compared with unstressed saline-treated group.

Moreover, significant decrease in SOD and GPx activity in the whole brain and all examined structures was observed in stressed mice receiving 0.5 mg/kg of nicotine in case of SOD (whole brain *P* < 0.01, cerebellum *P* < 0.001, hippocampus *P* < 0.001) and GPx (whole brain *P* < 0.01, cerebellum *P* < 0.001, cortex *P* < 0.001) in comparison with stressed saline-treated group. Whereas, nicotine at the dose of 0.2 mg/kg decreased SOD value only in the cerebellum (*P* < 0.01) and hippocampus (*P* < 0.05) as well as decreased GPx value in the cortex (*P* < 0.01) as compared with the stressed saline-treated group. Also, nicotine administered at the dose of 0.1 mg/kg decreased SOD value in the cerebellum (*P* < 0.05). In unstressed mice receiving 0.5 mg/kg of nicotine, significant dose-dependent decrease in antioxidant enzymes activity (SOD and GPx) in the whole brain and all examined structures was observed in case of SOD (*P* < 0.001) and GPx (*P* < 0.001) in comparison to unstressed saline-treated group. Whereas, nicotine at the dose of 0.2 mg/kg decreased SOD value only in the hippocampus and cortex (*P* < 0.001) as well as decreased GPx value (whole brain *P* < 0.001, cerebellum *P* < 0.001, hippocampus *P* < 0.05, cortex *P* < 0.001) as compared with the unstressed saline-treated group. Also, nicotine administered at the dose of 0.1 mg/kg decreased GPx value only in the cerebellum (*P* < 0.05).

The procedure of CUMS caused statistically significant increase in MDA concentration in stressed mice in comparison to unstressed control group (whole brain *P* < 0.001, cerebellum *P* < 0.001, hippocampus *P* < 0.001, cortex *P* < 0.01). Moreover, nicotine at the dose of 0.2 mg/kg increased MDA value in the whole brain (*P* < 0.05) and cortex (*P* < 0.001) as compared with the unstressed saline-treated group. Also, nicotine administered at the dose of 0.1 mg/kg increased MDA value only in the cortex (*P* < 0.05). The stressed animals, receiving the highest dose of nicotine (0.5 mg/kg) exhibited significant increase in MDA concentration in the whole brain (*P* < 0.001) and cortex (*P* < 0.01) in comparison to stressed saline group. The increase in MDA level in unstressed mice receiving nicotine at the dose of 0.5 mg/kg (whole brain *P* < 0.001, cerebellum *P* < 0.05, hippocampus *P* < 0.001, cortex *P* < 0.001) in comparison to unstressed saline group was also observed.

Table 3 further presents effects of the CUMS and also a subchronic administration of nicotine in stressed and unstressed mice on chosen markers of oxidative stress. Data are presented for TAS: in the whole brain (two-way ANOVA: condition effect [*F*(3,72) = 12.81, *P* < 0.0001], treatment effect [*F*(1,72) = 52.28, *P* < 0.0001] without interaction effect [*F*(3,72) = 0.47, *P* = 0.7071]) as well as in single structures as cerebellum (two-way ANOVA: condition effect [*F*(3,72) = 4.86, *P* = 0.0039], treatment effect [*F*(1,72) = 85.45, *P* < 0.0001] without interaction effect [*F*(3,72) = 0.04, *P* = 0.9902]), hippocampus (two-way ANOVA: condition effect [*F*(3,72) = 13.50, *P* < 0.0001], treatment effect [*F*(1,72) = 43.92, *P* < 0.0001] without interaction effect [*F*(3,72) = 0.32, *P* = 0.8111]), and cortex (two-way ANOVA: condition effect [*F*(3,72) = 11.89, *P* < 0.0001], treatment effect [*F*(1,72) = 24.13, *P* < 0.0001] without interaction effect [*F*(1,72) = 0.77, *P* = 0.5166]); SOD: in the whole brain (two-way ANOVA: condition effect [*F*(3,72) = 16.86, *P* < 0.0001], treatment effect [*F*(1,72) = 156.28, *P* < 0.0001] without interaction effect [*F*(3,72) = 1.49, *P* = 0.2239]) as well as in single structures as cerebellum (two-way ANOVA: condition effect [*F*(3,72) = 13.64, *P* < 0.0001], treatment effect [*F*(1,72) = 76.08, *P* < 0.0001] without interaction effect [*F*(3,72) = 1.12, *P* = 0.3477]), hippocampus (two-way ANOVA: condition effect [*F*(3,72) = 26.98, *P* < 0.0001], treatment effect [*F*(1,72) = 206.00, *P* < 0.0001] with interaction effect [*F*(3,72) = 7.49, *P* = 0.0002]), and cortex (two-way ANOVA: condition effect [*F*(3,72) = 23.41, *P* < 0.0001], treatment effect [*F*(1,72) = 222.95, *P* < 0.0001] with interaction effect [*F*(1,72) = 6.45, *P* = 0.0006]); GPx in the whole brain (two-way ANOVA: condition effect [*F*(3,72) = 16.41, *P* < 0.0001], treatment effect [*F*(1,72) = 482.20, *P* < 0.0001] with interaction effect [*F*(3,72) = 3.00, *P* = 0.0360]) as well as in single structures as cerebellum (two-way ANOVA: condition effect [*F*(3,72) = 53.77, *P* < 0.0001], treatment effect [*F*(1,72) = 1226.01, *P* < 0.0001] with interaction effect [*F*(3,72) = 13.30, *P* < 0.0001]), hippocampus (two-way ANOVA: condition effect [*F*(3,72) = 9.96, *P* < 0.0001], treatment effect [*F*(1,72) = 360.13, *P* < 0.0001] with interaction effect [*F*(3,72) = 2.80, *P* = 0.0460]), and cortex (two-way ANOVA: condition effect [*F*(3,72) = 37.62, *P* < 0.0001], treatment effect [*F*(1,72) = 709.90, *P* < 0.0001] with interaction effect [*F*(1,72) = 10.44, *P* < 0.0001]); MDA in the whole brain (two-way ANOVA: condition effect [*F*(3,72) = 19.10, *P* < 0.0001], treatment effect [*F*(1,72) = 161.52, *P* < 0.0001] without interaction effect [*F*(3,72) = 0.18, *P* = 0.9121]) as well as in single structures as cerebellum (two-way ANOVA: condition effect [*F*(3,72) = 7.93, *P* = 0.0001], treatment effect [*F*(1,72) = 602.74, *P* < 0.0001] without interaction effect [*F*(3,72) = 1.88, *P* = 0.1398]), hippocampus (two-way ANOVA: condition effect [*F*(3,72) = 33.00, *P* < 0.0001], treatment effect [*F*(1,72) = 36.13, *P* < 0.0001] without interaction effect [*F*(3,72) = 1.20, *P* = 0.3163]), and cortex (two-way ANOVA: condition effect [*F*(3,72) = 18.40, *P* < 0.0001], treatment effect [*F*(1,72) = 16.05, *P* < 0.0001] without interaction effect [*F*(1,72) = 1.63, *P* = 0.1888]).

**Table 3:** Effect of the CUMS and nicotine administered subchronically (day 15–27) in unstressed and stressed mice subjected to the CUMS on chosen parameters of antioxidant barrier and process of lipids peroxidation. Nicotine (0.05, 0.1, and 0.5 mg/kg) was administered s.c. 30 min before the CUMS procedure as well as to unstressed control mice, and additionally 30 min before the tests on the 28th day of the experiment

	Tissue	Non-stressed				Stressed			
		Saline	Nic 0.05	Nic 0.1	Nic 0.5	Saline	Nic 0.05	Nic 0.1	Nic 0.5
TAS	Brain	1.892 ± 0.194	1.824 ± 0.130	1.745 ± 0.180	1.520 ± 0.205***	1.588 ± 0.208**	1.524 ± 0.176	1.428 ± 0.1751	1.318 ± 0.086^#^
	Cerebellum	2.101 ± 0.321	2.013 ± 0.199	1.912 ± 0.229	1.868 ± 0.127	1.680 ± 0.165***	1.596 ± 0.1482	1.505 ± 0.132	1.485 ± 0.180
	Hippocampus	1.591 ± 0.162	1.514 ± 0.171	1.545 ± 0.123	1.283 ± 0.143***	1.363 ± 0.1586*	1.305 ± 0.170	1.266 ± 0.096	1.094 ± 0.179^##^
	Cortex	1.729 ± 0.238	1.689 ± 0.219	1.513 ± 0.114	1.351 ± 0.221***	1.467 ± 0.172*	1.425 ± 0.128	1.371 ± 0.163	1.215 ± 0.169
SOD	Brain	2.755 ± 0.376	2.668 ± 0.340	2.350 ± 0.246*	2.104 ± 0.177***	1.885 ± 0.197***	1.855 ± 0.197	1.763 ± 0.211	1.488 ± 0.249^#^
	Cerebellum	8.004 ± 1.244	7.179 ± 1.114	5.932 ± 1.059**	5.243 ± 1.374***	4.971 ± 1.330***	4.772 ± 1.239	4.022 ± 1.013	3.419 ± 0.965
	Hippocampus	14.37 ± 1.374	12.23 ± 1.242*	10.67 ± 1.297***	8.757 ± 0.903***	8.027 ± 1.369***	7.429 ± 1.547	7.091 ± 1.510	6.237 ± 1.399
	Cortex	14.39 ± 1.073	12.88 ± 2.007	10.66 ± 1.480***	9.168 ± 1.023***	7.945 ± 1.071***	7.229 ± 1.478	6.957 ± 1.530	6.133 ± 1.344
GPx	Brain	16.44 ± 1.082	14.74 ± 1.826	12.87 ± 1.945***	11.98 ± 2.220***	7.278 ± 0.880***	6.751 ± 1.419	6.235 ± 1.340	5.284 ± 1.212
	Cerebellum	37.33 ± 1.848	32.61 ± 2.050***	30.91 ± 2.761***	23.99 ± 2.670***	15.93 ± 2.486***	14.12 ± 2.377	12.11 ± 1.807^##^	11.38 ± 2.000^###^
	Hippocampus	63.72 ± 4.596	55.42 ± 8.690*	55.15 ± 5.797*	48.69 ± 6.643***	33.42 ± 5.207***	32.49 ± 4.113	30.51 ± 4.823	28.68 ± 4.958
	Cortex	66.19 ± 2.875	58.15 ± 5.296**	53.27 ± 4.342***	43.82 ± 5.937***	32.34 ± 3.755***	29.52 ± 3.937	27.53 ± 4.834	25.46 ± 4.078^#^
MDA	Brain	2.404 ± 0.514	2.814 ± 0.668	3.314 ± 0.701	4.874 ± 1.293***	5.614 ± 0.942***	5.631 ± 1.361	6.433 ± 1.373	7.713 ± 1.181^###^
	Cerebellum	2.339 ± 0.318	2.722 ± 0.442	2.986 ± 0.330	3.008 ± 0.411	5.766 ± 0.613***	5.928 ± 0.749	6.068 ± 1.010	6.955 ± 0.752^##^
	Hippocampus	1.462 ± 0.289	1.563 ± 0.214	2.074 ± 0.301	2.884 ± 0.331***	2.223 ± 0.790**	2.354 ± 0.550	2.466 ± 0.366	3.301 ± 0.391^###^
	Cortex	12.08 ± 1.751	14.92 ± 1.672	17.67 ± 2.920***	18.18 ± 2.414***	16.13 ± 2.337**	16.55 ± 2.522	18.46 ± 3.124	20.37 ± 2.222^##^

The values represent the mean ± SEM (*n* = 8–12 mice per group). *P < 0.05, **P < 0.01, ***P < 0.001 vs. unstressed, saline-treated group, ^#^ *P* < 0.05, ^##^ *P* < 0.001, ^###^ *P* < 0.001 vs. stressed, saline-treated group (Tukey’s post hoc test)

A post hoc analysis showed that the exposition to the CUMS protocol decreased the values of TAS (whole brain *P* < 0.01, cerebellum *P* < 0.001, hippocampus *P* < 0.05, cortex *P* < 0.05), activities of SOD (whole brain *P* < 0.001, cerebellum *P* < 0.001, hippocampus *P* < 0.001, cortex *P* < 0.001) and GPx (whole brain *P* < 0.001, cerebellum *P* < 0.001, hippocampus *P* < 0.001, cortex *P* < 0.001) with simultaneous increased in MDA concentrations (whole brain *P* < 0.001, cerebellum *P* < 0.001, hippocampus *P* < 0.01, cortex *P* < 0.01) in stressed mice as compared with unstressed control. Furthermore, subchronic treatment with 0.5 mg/kg of nicotine significantly increased TAS value in the whole brain (*P* < 0.001), hippocampus (*P* < 0.001), cortex (*P* < 0.001) in unstressed mice as compared with unstressed saline-treated group. Moreover, nicotine (0.5 mg/kg) decreased TAS value in the whole brain (*P* < 0.05), and hippocampus (*P* < 0.01) as compared with the stressed saline-treated group.

In stressed mice, significant decrease in antioxidant enzymes activity in animals receiving 0.5 mg/kg of nicotine was observed in case of SOD (whole brain *P* < 0.05) and GPx (cerebellum *P* < 0.001, cortex *P* < 0.05) in comparison to stressed saline-treated group, whereas nicotine at the dose of 0.1 mg/kg decreased GPx value (cerebellum *P* < 0.01) as compared with the stressed saline-treated group. Significant dose-dependent decrease in antioxidant enzymes activity (SOD and GPx) in the whole brain and all examined structures was also observed in unstressed mice receiving chronically 0.5 mg/kg of nicotine in case of SOD (*P* < 0.001) and in GPx (*P* < 0.001) in comparison to unstressed saline-treated group. Whereas, in these mice nicotine at the dose of 0.1 mg/kg decreased SOD value in the whole brain (*P* < 0.05), cerebellum (*P* < 0.01), hippocampus (*P* < 0.001), cortex (*P* < 0.001) as well as decreased GPx value in the whole brain (*P* < 0.001), cerebellum (*P* < 0.001), hippocampus (*P* < 0.05), cortex (*P* < 0.001) as compared with the unstressed saline-treated group. Also, nicotine administered at the dose of 0.05 mg/kg decreased SOD activity in the hippocampus (*P* < 0.05) and GPx activity in the cerebellum (*P* < 0.001), hippocampus (*P* < 0.05), and cortex (*P* < 0.01).

Concerning MDA level, stressed animals, receiving the highest dose of nicotine (0.5 mg/kg) exhibited significant increase in MDA concentration in the whole brain (*P* < 0.001), cerebellum (*P* < 0.01), hippocampus (*P* < 0.001), and cortex (*P* < 0.01) in comparison to stressed saline group. The increase in MDA level of unstressed mice receiving nicotine at the dose of 0.5 mg/kg (whole brain *P* < 0.001, hippocampus *P* < 0.001, cortex *P* < 0.001) in comparison to unstressed saline group was also observed. Moreover, nicotine at the dose of 0.1 increased MDA value in the cortex (*P* < 0.001) as compared with the unstressed saline-treated group.

## Discussion

Stress has been already defined as a non-specific response of the body to any change, and has been associated with a greater risk for different clinical conditions, including psychiatric disorders, such as depression and anxiety [35]. To better study the consequences of stress on behavior, animal models have been developed including the CUMS procedure. Briefly, in this model, rodents are exposed to a variety of relatively mild neither controllable nor predictable stressors (i.e., restriction, water/food deprivation, damp sawdust) intermittently, usually for 3–4 weeks [8, 9, 11, 26]. Studies in animals have shown that the CUMS model is able to evoke behavioral changes resembling clinical depression symptoms, such as locomotor activity impairment, reduced food or water consumption, and a decrease in responsiveness to rewarding stimuli [36]. Nevertheless, results from studies testing anxiety- or depression-related reactions induced by prior exposure to stress are quite ambiguous. While some authors have reported increases in anxiety and depression, others have found no effects or even a decrease in anxiety and/or depression-related responses after exposure to different kinds of stressors or to different models [26, 37, 38]. Possible explanation for the different effects observed might rely on the magnitude or kind of the stressor used.

The CUMS model presents good validity and has been broadly used to investigate some of the physiological and behavioral consequences of chronic stress [8, 9, 11, 26]. Thus, in the present study, animals were either unstressed or exposed for 4 weeks to an unpredictable chronic mild stress procedure and subsequently tested in the battery of behavioral paradigms, including anxiety- or depression-related in the EPM and FST tasks or cognitive effects in the PA paradigm. In this context, our results revealed that CUMS-exposed animals exhibited several behavioral alteration, resembling the symptoms of anhedonia and chronic human depression, anxiety disorders and the disturbances in memory formations, i.e., increased immobility time in the FST, decreased percentage of the time spent on the open arms and the percentage of open arm entries in the EPM test as well as decreased IL value in the PA test in stressed mice as compared to unstressed control.

It has been established that stress can produce behavioral and metabolic changes in exposed organisms, which can result in somatic and mental disorders. However, the exact neuronal mechanism behind the main effect of chronic stress exposure is still to be elucidated. These diverse effects include alterations of the catecholaminergic, opioidergic, serotonergic, and steroid systems in the brain, and may contribute to its pathophysiology [39, 40]. There has been very little research examining the cholinergic mechanisms of the CUMS-induced changes. Thus, increases in cholinesterase expression in the hippocampus following CUMS have been reported, whereas reductions in cholinesterase activity have been described in the cortex, hypothalamus, and striatum (but not hippocampus) [41]. Also, a paper has documented reductions in muscarinic cholinergic receptors in the hippocampus and cortex, but not hypothalamus [42]. Changes in cholinergic function following CUMS may reflect changes in cognitive function from stress and depression, but further work is required in this domain to understand further the influence of CUMS on the cholinergic pathways. This is particularly important since there is a body of evidence which has indicated that dysfunction of the cholinergic system may be present in major depression, such as impaired acetylcholinesterase activity or increased activity at central nicotinic receptors [43]. In the context of stress-induced behavioral changes and the influence of cholinergic pathways, in the present study we demonstrated memory impairment and depression- or anxiety-like behaviors induced by the CUMS, nicotine and their interactions in mice. As such, nicotine, after an acute or repeated administration, reversed the depression-like and anxiogenic effects of the CUMS in the FST and EPM, respectively, as well as memory disturbances measured in the PA paradigm. Moreover, in the FST, in stressed mice submitted to the CUMS protocol, significant decrease in the antidepressant effects of nicotine has been observed. In this animal model, metyrapone, a corticosterone synthesis inhibitor, diminished the depression-like effect of stress suggesting the influence of glucocorticosteroids and the HPA axis in stress-induced anhedonia.

Nicotine is the psychoactive substance responsible for the addictive use of tobacco, which results in numerous harmful health effects and continues to be the leading cause of preventable death [44]. It has been reported that addicted tobacco users suffer from nicotine-induced cognitive impairments, as well as modulated mood such as anxiety- and depression-like symptoms [45]. Furthermore, it has been suggested that brain regions such as the medial prefrontal cortex, for which nicotine-induced effects have been demonstrated, are concurrently involved in the development of stress-induced working memory impairments and anxiety [46, 47]. However, there are only few studies investigating the specific effects of nicotine as a stressor, particularly those on cognitive function or anhedonia-related mood disturbances [48]. In some rodent models, CUMS or acute stress has been shown to aggravate the behavioral and neuronal effects of nicotine [49]. However, some antagonistic effects of subsequently administered nicotine against the behavioral (cognitive) and neuronal impairments caused by stress have been reported in some rodent models [50–52]. With respect to the interactions between nicotine, stress, and anhedonia-related syndromes, it has been shown that both nicotine and CUMS enhanced each other’s unfavorable effects on behavioral function including those on the working memory- and anxiety-related behavioral alterations in the Y-maze and EPM tests [53]. Therefore, the relationship between stressors such as the CUMS and nicotine remains controversial (i.e., *antistress* effects of nicotine have also been reported depending on the conditions) [50].

It still remains controversial whether nicotine actually reduces stress in humans and animals. Thus, the regulatory pathways mediating behavioral and metabolic response to stress and/or nicotine have not been clearly demonstrated so far. One of the limiting factors is that the pattern of these changes strongly depends on the kind of the stress stimulus. It is of interest to understand the behavioral effects of nicotine that might explain ongoing tobacco use, as it has been shown that cigarette smoking can diminish anxiety and relieve stress in humans, which are likely attributable to the nicotine as a psychoactive component of cigarette smoke [54]. The most striking feature of the recent study is that nicotine, after its chronic administration, attenuates stress-induced decrease in locomotor activity and in plasma amino acid concentrations [50, 55]. Numerous human studies have demonstrated an increase in smoking rate among smokers exposed to stress, and such a paradoxical behavior is believed to reduce the subjective feeling of stress-related tension [15, 56]. At the same time, nicotine dependence may be associated with mood liability and anxiety, leading to increased feelings of stress in many regular smokers. Actually, smoking under stress lead smokers to stress levels that are comparable to nonsmokers, suggesting that acute nicotine withdrawal increases stress, and nicotine reinstatement relieves feelings of stress [57].

Anxiety is thought to be a negative emotion caused by many kinds of stress. In this field, the EPM task has become one of the most popular animal paradigms also used in our study. In this test, the anxiety-like behavior (i.e., decreased percentage time spent in the open arms and open arms entries) is potentiated by prior exposure to a variety of stressors [58], as confirmed by our data. Nicotine has been shown to affect anxiety in different ways in animal studies. In rodents, nicotine can be anxiolytic, anxiogenic, or have no effect on anxiety, depending on the dose used, the route of administration, the behavioral test performed and the time of testing after nicotine administration [24, 30, 59, 60]. However, there is less reports related to the effects of nicotine on stress-enhanced anxiety [55]. Our study has demonstrated that nicotine administered acutely (at the active dose) or subchronically (at the inactive dose), decreased the anxiety-like effects of stress in mice after the CUMS protocol. Concerning depression-like effects measured in the FST, also commonly used in rodents, nicotine has been shown to provoke antidepressive action, i.e., decrease in immobilization time [61] also confirm in our study in non-stressed mice. Similar to anxiety, our data revealed that nicotine can diminish depression-like effects of chronic mild stress. Moreover, in stressed mice submitted to the CUMS protocol, significant decrease in the antidepressant effects of nicotine by itself has been observed suggesting the reciprocal influence of stress and nicotine. Although the precise mechanism of such an effect is not clear, it can be speculated that it involves nicotinic receptor-mediated modulation of the responses of the HPA axis to stress.

Concerning neuronal mechanisms of above-mentioned interactions, differential pathways activation induced by stress are reported in different studies, in terms of the nature of the stress and the intensity of the stimulus [62, 63]. There is a general agreement that both stressor stimuli and nicotine activate central nervous system [64–66]. Certain forebrain regions seem to be pivotal in gating the stress-induced inputs to the paraventricular nucleus of the hypothalamus (PVN). Medial prefrontal cortex, central amygdala, bed nucleus of the stria terminalis, and hippocampus, as major regulatory inputs to the PVN, have been implicated in the induction of the enhanced anxiety state induced by stressors or anxiogenic drugs [67–69]. In a recent paper, nicotine was shown to reduce the restraint enhanced anxiety-like behaviors and also the restraint-induced c-Fos expression in several brain regions implicated in stress and anxiety [55]. In general, stress can produce an array of hormonal, autonomic, and behavioral responses. Moreover, chronic stress exposure and resulting dysregulation of the HPA axis develops susceptibility to variety of neurological and psychiatric disorders including anxiety- and depression or memory disturbances. Accordingly, it has been reported that nicotine administration during restraint stress enhances the increase in plasma corticosterone, as compared to the responses induced by either factor alone. On the contrary, in one study the restraint-induced increase in plasma corticosterone was not affected by nicotine [55]. The disparity of nicotine’s effect on stress-induced responses indicates that stress-induced anxiety-like behavior might be dissociated with HPA axis activation. Considering this kind of dissociation between anxiety-like behavior and the HPA axis activation [70], it is suggested that nicotine might selectively modify the neuronal circuits related to behavioral changes rather than hormonal response to stress. This effect, which is independent on the plasma levels of corticosterone, may be associated with the reduction of above-mentioned stress-induced c-Fos expression in certain brain regions involved in stress or anxiety rather than affecting HPA axis hormone responses, as already stated.

In light of the data which have shown that nicotine can diminish the memory impairment caused by a chronic stress, it has been reported that modifications in working memory (both ameliorations and impairments) can occur due to even slight changes in prefrontal dopamine levels [71]. Stress also activates two dopaminergic pathways, such as the mesolimbic and mesocortical systems which are involved in the regulation of adaptive processes. It is considered that experiencing stressogenic situations change the metabolism and release of dopamine in the mesolimbic system. Moreover, repeated exposure to stress can change the ability to react to subsequent stressors, leading to disturbances in the function of the mesolimbic system which is significantly related to the central action of nicotine. Exposure to a single, unexpected, and uncontrolled aversive stimulus leads to inhibition of dopamine release and disturbances in the reaction to rewarding and aversive stimuli [72]. Therefore, the working memory-related behaviors in the nicotine-treated mice may be correlated with alterations in nicotinic cholinergic receptor (nAChR)-mediated prefrontal dopamine release, which is controlled by specific nAChR subtypes (e.g., alpha7 nAChRs) [73]. In addition to dopamine release, the release of other neurotransmitters such as glutamate has been implicated in the nicotine-induced working memory formation [74]. In studies on mice or rats, it has been proposed that acute or chronic stress disturbs hippocampal-dependent memory formation [75] and reverses long-term potentiation (LTP) induction, which is the basis of synaptic plasticity and memory formation [76]. The dentate gyrus (DG) of the hippocampus shows pronounced functional and morphological changes in response to the CUMS. Animals exposed to stress display atrophy of CA3 pyramidal neurons, reduced neurogenesis in the DG, and alterations in hippocampal apoptosis [77–79]. The increases in serum corticosterone observed in animals submitted to the CUMS protocol corroborate previous evidence [80], and suggest that, as already described, chronic stress exposure effectively activates the HPA axis, what is possibly responsible for the decreases in neuronal migration observed in the hippocampus, as indicated by previous studies [46, 80]. This effect can explain the memory dysfunction after the CUMS protocol used in the current experiments. It is well established that nicotine causes an above-stated increase in the secretion of adrenocortiotropic hormone (ACTH) and corticosterone in human and other animals [81]. Thus, mesolimbic dopaminergic neurons possess corticosteroid receptors [82] and there are functional changes in nAChRs and glucocorticoid receptor in the brain. From this evidence and these results [83], it can be speculated that functional changes in nAChRs and glucocorticoid receptor in the brain may be induced by nicotine treatment and/or psychological stress in terms of cognition and anhedonia.

Taking into account our biochemical analysis, it has been already described that the brain monoamine or cholinergic and antioxidant defense systems interact closely with the HPA axis and can be implicated in the acquisition and consolidation of psychological and physical long-term stress responses [84, 85]. Chronic stress has been shown to increase the vulnerability of different brain regions, particularly the hippocampus, by altering the antioxidant defense capacity. As already stated, stress is an unavoidable life experience, and chronic stress has been related with deregulation of many biological and physical processes. Among them, increased oxidative stress within the central nervous system could correlate with impairments in brain function within behavior as well as control on other organs [86]. Therefore, we examined the effects of CUMS on TAS and key antioxidant enzymes of brain with special impact on the structures involved in cognitive functions, e.g., prefrontal cortex and hippocampus. Additionally, as SOD catalyzes dismutation of superoxide into hydrogen peroxide and then GPx catalyzes reduction of H_2_O_2_ into hydrogen and water, their inappropriate effectiveness causes production of hydroxyl radical (OH^⋅^). The radical is highly reactive although short-lived. However, it plays a crucial role in the process of lipids peroxidation. Stress has been well documented to induce alterations in antioxidant barrier as well as promote oxidative stress [87]. Our studies are along with those findings. We have confirmed involvement of CUMS-provoked induction of oxidative stress within brain and its particular structures as our results have proven decrease in TAS in stressed mice, decrease in antioxidant enzymes SOD and GPx as well as increase in concentration of MDA.

Literature data show that nicotine provokes cognition-enhancing and memory improving effects [21]. However, molecular studies prove that nicotine induces oxidative stress within liver, kidneys, heart and brain of experimental animals in vivo [88] and in vitro in cell cultures study [89]. The liver and kidneys are associated with nicotine metabolism while heart and brain are targeted organs of its action. The results of our previous study support the thesis that an acute nicotine treatment induces oxidative stress within brain structures responsible for learning and memory processes [21]. These experiments along with the present data showed significant increase in concentration of MDA as well as suppression of antioxidant enzymes (SOD and GPx) activities after nicotine administration within all examined brain structures and in the whole brain tissue, like in the case of CUMS in stressed animals. As such, the present study strongly confirmed pro-oxidative effect of nicotine administered acutely and subchronically and can prove that nicotine potentiates cortisol-induced oxidative stress on the level of total antioxidant status and antioxidant enzymes activity as well as increase in peroxidation of lipids. People in stress often smoke more as they feel more relaxed then. It is a strictly psychological impression, because in fact nicotine activates HPA axis on the stage of ACTH excretion and therefore increases the level of cortisol liberated to blood stream [90]. Additionally, our study further confirmed increase in oxidative stress parameters after acute as well as subchronic nicotine administration in stressed mice submitted to the CUMS protocol, i.e., decreased TAS, SOD, and GPx activity and increased MDA concentration. It is worth noting that several studies suggested that different results in antioxidant barrier status of tissues of animals’ exposure to chronic stress and/or nicotine may come from differences in protocols used for the induction of stress (different type of stress) as well as the applied animal model [17].

To sum up, acute or chronic stress is one of the most validated animal models for generating the depression-like symptoms observed in humans [91]. Animal models of CUMS represent valuable tools to investigate the behavioral, endocrine, and neurobiological changes underlying stress-related psychopathologies, such as anhedonia and memory disturbances, a core endophenotype of human depression. The present study was aimed at investigating the anxiety- and depression-related action as well as cognitive disturbances in mice exposed to the chronic stress model. To this purpose, mice were subjected to 4 weeks of chronic unpredictable stressful stimuli, after which the animals were submitted to a behavioral test, i.e., the FST, EPM and PA paradigm. Nicotine, after an acute and subchronic administration decreased stress-induced depression- and anxiety-like effect as well as memory deficit in mice indicating the relationship between stress and nicotine. Our study contributes to the understanding of the mechanisms that are possibly involved in the biological basis of central stress-induced disorders such as anxiety, depression, and other neurodegenerative conditions including memory disturbances. The present findings also support the hypothesis that neurotransmitter intervention in oxidative damage should be considered as a possible mechanism of pro-oxidative stress- or nicotine-induced alterations; however, understanding of its precise mechanisms warrants further studies required to explore these integrated dual mechanisms operating during stressful conditions.

Thus, elucidating how monoaminergic and cholinergic systems interact with one another during the development of anhedonia-like states will increase our knowledge of the pathogenesis of the disease and nicotine-stress interactions on the basis of the development of nicotine dependence and ongoing tobacco abuse.
